# Relationships among Energy Price Shocks, Stock Market, and the Macroeconomy: Evidence from China

**DOI:** 10.1155/2013/171868

**Published:** 2013-04-09

**Authors:** Rong-Gang Cong, Shaochuan Shen

**Affiliations:** ^1^School of Economics and Management, North China Electric Power University, Beijing 102206, China; ^2^Centre for Environmental and Climate Research (CEC), Lund University, 223 62 Lund, Sweden; ^3^State Key Laboratory Breeding Base of Green Chemistry Synthesis Technology, College of Chemical Engineering and Materials Science, Zhejiang University of Technology, Hangzhou 310014, China

## Abstract

This paper investigates the interactive relationships among China energy price shocks, stock market, and the macroeconomy using multivariate vector autoregression. The results indicate that there is a long cointegration among them. A 1% rise in the energy price index can depress the stock market index by 0.54% and the industrial value-adding growth by 0.037%. Energy price shocks also cause inflation and have a 5-month lag effect on stock market, which may result in the stock market “underreacting.” The energy price can explain stock market fluctuations better than the interest rate over a longer time period. Consequently, investors should pay greater attention to the long-term effect of energy on the stock market.

## 1. Introduction

Since 2002, the global crude oil price has risen rapidly. In July 2008, price of both WTI crude oil and Brent crude oil went beyond $140 per barrel. This results in fluctuation in the international market, causing the domestic oil price to increase, which in turn pushes the energy price up for coal and electricity [1]. As a result, the nationwide retail commodity price index also raises to some extent. All of these factors have a profound effect on China's macroeconomy. In the meantime, owing to appreciation of the China currency (RMB), and in anticipation of expansion in the Chinese economy during the Olympics Games, the Chinese stock market has steadily increased since 2006, which, as a consequence, has attracted investors world wide. As a result, observing whether the shocks in energy price are transmitted to the stock market will receive considerable attention from investors. It might be argued that this relationship reflects the efficiency of the Chinese stock market.

Extant research includes analyses of relationships between energy price, a country's macroeconomics, and its stock market. Research on the relationship between energy price and the macroeconomy is mainly focused on the relationship between crude oil price and GDP/GNP and the channels through which crude oil price influences the macroeconomy. In the former, the majority of scholars have found a reverse relationship between oil price and GDP/GNP which meant that rising oil price would depress GDP/GNP [2–4]. In the mid-1980s, however, the linear reverse relationship became not so evident. Various scholars studied the asymmetric relationship between oil price and macroeconomic activities [5–7]. Lardic and Mignon [8] found that while standard cointegration is rejected, there is evidence for asymmetric cointegration between oil prices and GDP in the major European countries. In recent years, scholars measured the elasticity of GNP to oil price [9]. In the latter, it is generally assumed that oil price influenced the macroeconomy at the demand and supply levels. As to the demand level, on the one hand, the rise of oil price can cause inflation [10] and suppress consumption [11, 12] and investment [13, 14]. If the Central Bank cannot increase the currency supply, the interest rate may tend to rise, which may influence the aggregate demand [15, 16]. On the other hand, the rise in oil price may transfer wealth from oil-importing countries to those exporting. This may deteriorate the purchasing power of oil-importing countries and affect their international trade [17]. As regards to the supply level, as oil is a major material for production, price increases will raise production costs and cause industries to scale-down or transfer to low energy-intensive industries [18]. This, in-turn, may affect production and increase unemployment [14, 19]. Meanwhile, the rise of production costs may result in cost-driven inflation. If the cost increase is transferred to industries downstream, their production cost will increase. So any rise in oil price, influences various industries in different ways. Fan et al. [20] researched the impact of rising international crude oil price on China's GDP, investments, consumptions, import, export, and so on. 

As shown previously, scholars have extensively researched the relationship between oil price shocks and a country's macroeconomy. But there is relatively little research on the relationship between oil price shocks and the financial markets—the exception being those that focused on developed countries. For example, Jones and Kaul [21], based on a standard cash flows/dividends valuation model, researched the stock market of US, Canada, Japan, and England. They found that the change of oil price had a decisive effect on the four countries' real stock returns. Sadorsky [22] identified that oil price, and its volatility, played an important part in explaining the real stock returns. The movement of oil price explained more than interest rates for the forecasting variances. Papapetrou [23] researched the Greek stock market. Likewise, he also found that oil price played an important component in explaining stock price movements, and positive oil price shocks suppressed real stock returns. Ewing and Thompson [24] also examined the cyclical relationships among industrial production, consumer prices, unemployment, and stock prices using time series filtering methods. 

Several scholars showed that oil price and its shocks influenced various industries differently [25]. A common held view is that shocks are beneficial for oil companies upstream, yet has an adverse effect on companies downstream and other industries. For example, Huang's research [26], based on correlative coefficient method and a VAR model, used the S & P 500 index, twelve industries' stock price indices, and three oil company stock prices. He found that crude oil future returns had significant abilities to explain oil companies' stock returns, which could be seen as their lead index, but had little effect on the total market. Faff and Brailsford [27] used an enlarged market model to research several industries returns in the Australian stock market. They found that oil price had an effect on stock prices. They also found that the oil and gas industry and diverse resources industry had positive sensitivities, while papermaking, packing, and transportation industry had negative sensitivities. Using Johansen cointegration test, Hammoudeh et al. [28] found that one-month to four-month WTI oil future price shocks explained oil extracting, refining, and marketing companies' stock price movement. Sadorsky [29] took Canadian companies as an example. Using the stock market index, energy price, interest rates, and exchange rates as explanatory variables, he found that the rise of the stock market index and oil price had a positive effect on oil companies' returns, while the rise of interest rates and exchange rates had a negative effect. Lanza et al. [30] used VAR/VECM models to research the relationships among six large oil companies, various stock markets, and the spread of crude oil future and spot price. They found that the greater the spread, the higher the oil companies' stock prices.

In summary, it can be stated that there are relationships among oil price, macroeconomy, and the stock market, which have been tested in several developed countries. Whether these relationships exist in China is the focus of this paper. As the Chinese economic dependency on energy increases, any rise in energy price has a significant effect on the macroeconomy. In essence, the stock market is a virtual economy. To know whether it is affected by energy price, this paper has focused on the Chinese Shanghai stock market using a VAR model. Based on impulse response functions and forecasting variance decomposition, it analyzes the interactive responses among several economic variables and energy price.

The paper is structured as follows. We begin with a brief introduction of the VAR model and then describe the data in Section 2.  Section 3 presents the empirical results. In Section 4, we discuss the results within the context of the Chinese situation. Next, we conclude with a summary and propose suggestions in Section 5. Finally, possible future work is presented.

## 2. Methodology and Data

### 2.1. Methodology of VAR Model

We have selected to use a vector autoregression (VAR) method [31]. Since VAR model requires all variables in the system to be stationary, a unit root test is initially completed. Here we choose ADF (augmented Dickey-Fuller test) method. Take oil price series as an example. The fundamental principle of ADF test is

(1)       ΔOILt=γOILt−1+∑i=1pβiΔOILt−i+ut,t=1,2,…,T  no  constant  or  linear  time  trends,ΔOILt=γOILt−1+a+∑i=1pβiΔOILt−i+ut,          t=1,2,…,T  constant,ΔOILt=γOILt−1+a+δt+∑i=1pβiΔOILt−i+ut, t=1,2,…,T  constant  and  linear  time  trends.

The letter “*a*” is a constant. *δt* is a linear trend function. Δ represents differentiation. *u*_*t*_ ~ i.i.d. *N*(0, *σ* ^2^) which form is chosen depends on a series graph. *p* is the optimum lag order, which depends on AIC (Akaike Information Criterion). The original hypothesis and the alternative hypothesis can be written as

(2) $$\begin{array}{c} H_{0}:\gamma =0,\\ H_{1}:\gamma <0. \end{array}$$

The original hypothesis for oil price time series has a unit root which is a nonstationary series. The alternative hypothesis is the oil price time series that does not have unit roots, which is a stationary series. As to nonstationary time series, another stationary test is needed. If the test shows that the first-order differentiation is stationary, the series is named as I (1); otherwise the second differentiation should be tested.

IP_*t*_,  OIL_*t*_,  *r*_*t*_, and STOCK_*t*_ stand for industry production, oil price, nominal interest rates, and stock market index, respectively. *y*_*t*_ = (IP_*t*_, OIL_*t*_, *R*_*t*_, and  STOCK_*t*_)′. The VAR model can be constructed as follows:

(3) $$\begin{array}{c} y_{t}=A_{1}y_{t-1}+A_{2}y_{t-2}+\cdots +A_{p}y_{t-p}+\varepsilon_{t}\;t=1,2,\ldots ,T, \end{array}$$

where  *p* is lag orders, which is determined by AIC and SC information criterion. *T* is the size of the sample. *A*_1_, *A*_2_,…, *A*_*p*_ and  **B**  are parameter matrices. *ε*_*t*_ are random disturbances which can correlate in the same time but cannot correlate with their lag variables and the variables on the right of the functions. 

 If IP_*t*_,  OIL_*t*_,  *R*_*t*_,  and  STOCK_*t*_  can be tested as the same number of the unit roots, which can be assumed as I (1), we can use JJ method to test whether there are cointegration among the series. In other words, whether there are long-term stable relationships among nonstationary variables.

 Based on VAR model, we can also use impulse response functions and forecasting variance decomposition to explain the model established. Impulse response functions can be used to test the effect of a standard variance shock on the endogenous variables and their future values. Its fundamental rationale is as follows.

 If *L* is defined as a lag operator, *Lx*_*t*_ = *x*_*t*−1_, it can be derived from (3) as follows:

(4) (Ik−A1L−⋯−ApLp)yt=εt,yt=(Ik−A1L−⋯−ApLp)−1εt,=(Ik+C1L+C2L2+⋯)εt.

The first variable of *y*_*t*_ (industry production) can be written as

(5) $$\begin{array}{c} {\text{IP}}_{t}=y_{1t}=\sum_{j=1}^{4}\left(c_{1j}^{\left(0\right)}\varepsilon_{jt}+c_{1j}^{\left(1\right)}\varepsilon_{jt-1}+\cdots \right)\;t=1,2,\ldots ,T, \end{array}$$

where  *c*_1*j*_^(*q*)^ is the first row and first line element of *C*_*q*_, which can be represented as *c*_1*j*_^(*q*)^ = ∂IP_*t*+*q*_/∂*ε*_*jt*_. It shows at period IP_*t*+*q*_'s response to a shock of  *y*_*jt*_  (IP_*t*_,  OIL_*t*_,  *r*_*t*_,  or STOCK_*t*_) at the condition that other variables keep constant. We refer to it as the impulse-response function, which is similar to the shock multiplier effect analysis. As there are correlations in the same period among various functions' random variances in VAR model, it needs to construct an orthogonal matrix to transform shocks from correlation in the same period to noncorrelation. There are many methods to accomplish this goal. Here we used general impulse method [32] which does not depend on the variables' orders in VAR model.

 Variance decomposition decomposes the forecasting variances by various variables shocks. We can use it to estimate the importance of various structural shocks. Its fundamental logic is as follows: take industrial production as an example. It can be known from (5) that what is in the brackets is the total *ε*_*j*_'s effect on IP_*t*_ up to now. If it can be assumed that there are not series correlations among *ε*_*j*_, the variances are

(6) E[(c1j(0)εjt+c1j(1)εjt−1+c1j(2)εjt−2+⋯)2]  =∑q=0∞(c1j(q))2σjj j=1,2,…,4.

Additionally, if there are no correlations among disturbances in the same period the variance of IP_*t*_ is the sum of four variances above mentioned as follows:

(7) $$\begin{array}{c} \text{var}\left({\text{IP}}_{t}\right)=\sum_{j=1}^{4}\left\{\sum_{q=0}^{\infty }{\left(c_{1j}^{\left(q\right)}\right)}^{2}\sigma_{jj}\right\}\;t=1,2,\ldots ,T. \end{array}$$

Because the variance of IP_*t*_ can be decomposed into four irrelative effect, we can define the criterion as follows to measure the contribution of various disturbance to the variance of IP_*t*_:

(8) RVCj→1(∞)=∑q=0∞(c1j(q))2σjjvar(IPt)=∑q=0∞(c1j(q))2σjj∑j=14∑q=0∞(c1j(q))2σjj j=1,2,…,4.

The relative variance contribution (RVC) measures the effect of the *j*th variable on IP_*t*_. This paper decomposes each endogenous variable's shock to four parts related to every function's disturbance, which can be used to know the relative importance of various shocks to endogenous variables in the model. Comparing the importance varying with time, we also estimate the lag of the variable's effect.

### 2.2. Data Sources

 In this paper, we do not choose oil price data. Drawing upon lessons from Papapetrou [23], we choose domestic energy price index. Different from commodities price index for fuel that he chose in his paper, we choose purchase price index for fuel power. Since Chinese oil prices are not in accordance with international markets, we do not choose oil spot price or future price in international markets. Interestingly, though, since June 2006, the home refined oil price started to adjust in accordance with the movement of oil price in international market. Based on Jiao et al.'s [33] research, the domestic oil price responded 78.4% to the change of crude oil cost, and it had a four-month lag. Some of the international oil price shocks were absorbed by oil companies. The purchase energy price index can accurately reflect the domestic oil price in a timely manner.

 Considering that the energy price has a close relationship to industry, we chose industry value adding as a proxy for the macroeconomy. As for the stock market, we selected the monthly Chinese Shanghai stock market index and considered the bonus and stock dividend. According to traditional financial theory, it is generally assumed that the stock price is equal to the discount of stock's future cash. So interest rates are important to stock price. Following Sadorsky [22] and Papapetrou [23], we introduce interest rates to dynamic analysis. Interest rates can be divided into nominal interest rates and real interest rates. This paper chooses the latter. We have selected one year to mature a lump deposit for total withdrawal as a representative and eliminate the interest tax. With exception of the interest rate data sourced from the website of Bank of China, all the data is from the National Bureau of Statistic of China. With due consideration of availability of information, we selected monthly data between January 2000 and December 2010. The industry value-added data and the energy price data are both adjusted by Census X12 method and discounted by consumer price index. All the variables are in logarithmic form. As interest rates are in percentages, we define the logarithm of it as log⁡(1 + *r*/100).

## 3. Results and Analysis

### 3.1. The Stationary Test of Data

In Table 1, ADF test shows that industrial value added has two unit roots, and other variables have a unit root.

### 3.2. The Determination of Lag Orders

 Through testing on 1 lag order to 5 lag orders, AIC and SC can be obtained from Table 2.

Through test iterations, we find when the largest lag order equals to three, the AIC reaches the minimum. SC reaches the minimum when the lag order equals to one. So LR test is chosen as tradeoff. The original hypothesis test is the largest lag order equals to one. The testing statistic is as follows:

(9) LR=−2∗(l1−l3)=−2∗(774.352−815.9662)=83.2284,

where  *l*_1_ and *l*_3_ are the whole log likelihood function values when *p* equals to one and three, respectively. In the original hypothesis, the statistic has gradually conformed to *χ* ^2^ distribution whose degrees of freedom are thirty-two. The accompanied probability is  1.9∗10^−6^. So we chose a three lag order VAR model, because the original hypothesis is rejected.

### 3.3. The Estimation and Test of the Model

 To give the variables the same integration order, we choose the first lag order to the third lag order values of OIL, *R*, STOCK, and  *d*IP  as an alternative of  IP  as endogenous variables and ordinary least squares method to estimate the model. The results are as follows:

(10) (dIPtOILtRtSTOCKt) =(−0.92∗−0.1750.730.12∗−0.0561.27∗−0.2230.0048−0.11∗0.118∗1.17∗0.0170.36−0.26−1.481.056)(dIPt−1OILt−1Rt−1STOCKt−1) +(−0.69∗0.191.750.006−0.02−0.35∗−0.1630.008−0.079∗−0.14−0.067−0.005−0.030.231.82−0.09)(dIPt−2OILt−2Rt−2STOCKt−2) +(−0.15−0.122−2.8∗−0.2∗−0.020.040.29−0.045−0.010.017−0.19−0.0150.280.180.470.03)(dIPt−3OILt−3Rt−3STOCKt−3) +(1.05∗0.43∗0.06−0.67),

where *  stands for coefficients that are significant.

As four series in the model are I (1) series, the condition for cointegration is satisfied. The lag order is determined to 2. Johansen cointegration test result shows that there is a cointegration relationship among four series above which demonstrates that there is a prolonged stationary relationship among macroeconomy, energy price, and the stock market (Table 3).

 The cointegration can be expressed as

(11) $$\begin{array}{c} d\text{IP}=-0.037^{*}\text{OIL}-0.14R-0.02^{*}\text{STOCK}+0.36. \end{array}$$

We can see from the previous equation that in the longer term, the 1% rise in energy price will have an adverse effect of 0.037% on industrial value adding and an adverse effect of 0.54% on the stock market.

 Testing using VAR model obtains the following results (Table 4).

Generally, it is difficult to analyze the dynamic relationships among variables based on the estimated parameters in the VAR model. Consequently, we use impulse-response function and variance decomposition to analyze the interactive relationships among them.

### 3.4. Impulse-Response Function and Variance Decomposition

**Figure 1:**
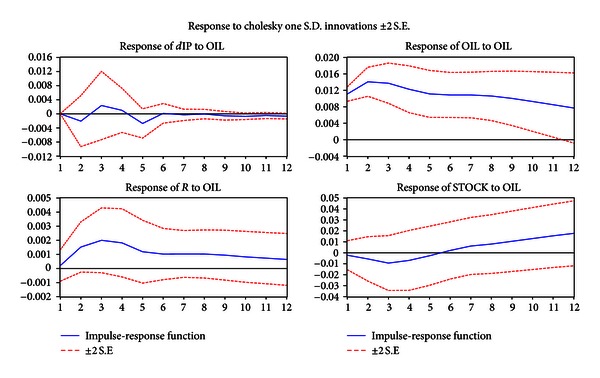
Generalized impulse responses to one S.D. shock for energy price changes. Note that the horizontal axis is the period. The vertical axis is the explanation level of dependent variables to independent variables. In the model, we fix the periods at 12 months.

Figure 1 shows the impulse-response function curves simulated by analytic method, based on VAR model. 

 We consider the response of four variables to one S.D. (standard deviation) innovation of energy price.

 For one standard deviation innovation of energy price (1.1% of energy price), interest rate apparently respond. In 3 months, it rises to the peak (0.2%). In 5 months, the effect keeps stable, which represents the level of interest rate with a 1% stable increase.

 One standard deviation innovation of energy price to the growth rate of industrial value adding has the greatest adverse effect (0.2%) in 2 months. Also, there is a shock following. But it has a negative convergence trend in the long term.

 As for one standard deviation innovation of energy price, the stock price responds adversely to its peak in 3 months (1%). But in 6 months, the adverse effect disappears.

**Figure 2:**
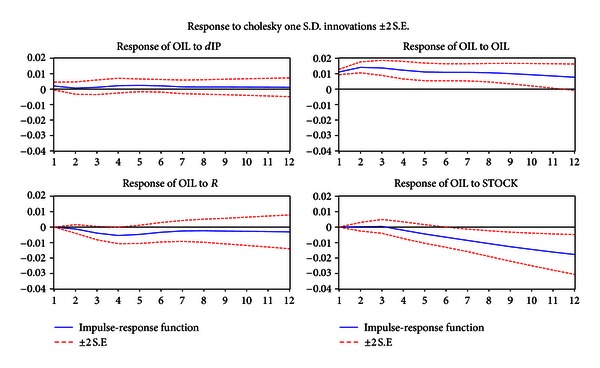
Generalized impulse responses of energy price to one S.D. shock for other variables changes. Note that the horizontal axis is the period. The vertical axis is the explanation level of dependent variables to independent variables. In the model, we fix the periods at 12 months.

 Next, we consider the effect of four variables' shocks on the energy price index (Figure 2).

 One standard deviation shock for interest rate shows that energy price is affected most adversely in four months and remains so for a long time. The residual variances are still half of its peak in a year.

 Energy price has little response to one standard deviation shock of industrial value adding.

 The stock price rises 5%. In three months the energy price has a downward trend. Twenty months later, the adverse effect has reached its minimum value (2%).

 We decompose the variance to stock market price based on VAR model and analytic method (above mentioned). The results are as follows.

**Figure 3:**
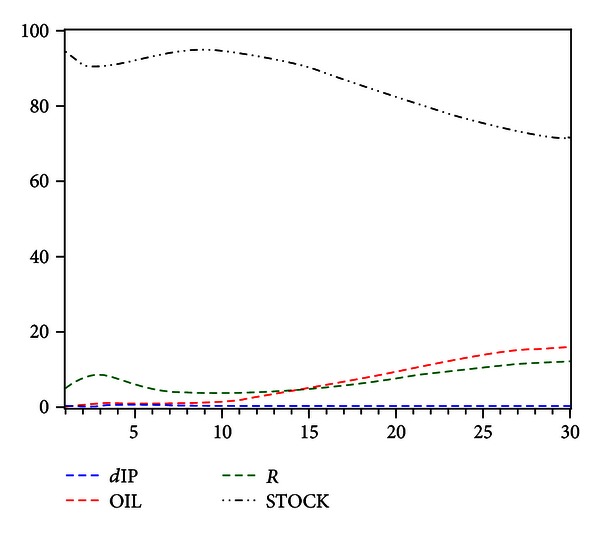
Variance decomposition of STOCK.

 In Table 5, the first and seventh columns are the periods that are set to a maximum of twenty due to limited space. The data in the S.E. (standard error) column are the forecasting variance of STOCK in various periods, which are caused by the change of the present or future value. *d*IP, OIL, *R*, and STOCK are the contribution of the innovations to forecasting variance, respectively, which sum to 100.  Figure 3 is the variance decomposition where the periods are prolonged to thirty.

 It can be seen from Figure 3 that, at the first month, the stock price can only explain 94% of its forecasting variance. The energy price can explain 0.14%, the industrial value-added can explain 0.4%, and the change rate of interest rate can only explain 5.03%. In fifteen months, the importance of energy price exceeds interest rates. At the twentieth month, the interest rates and energy price can explain 7.7% and 9.56%, respectively, while the industrial value added can only explain 0.24%.

## 4. Discussion 

### 4.1. There Is a Cointegration among Stock Market, Macroeconomy and Energy Price

 In this paper, we find that there is a long-term stable correlation among the stock market, macro-economy and energy price. Stock market is often said to be the barometer of one's macroeconomy, which means that they should have a close relationship. But it is a controversial issue in China. Chinese scholars generally have two views. One is that the stock market has a weak positive correlation with the macroeconomy. The other perspective is that the correlation is not significant or is negative. Scholars holding this latter view believe that the Chinese stock market aims at financing but not investing. The stock market can not evaluate the value of companies correctly. Therefore, the stock market is not the barometer of the Chinese macroeconomy. Based on a capital asset pricing model, the real value of stock equals to the discount of its future cash flows. As a result, the value of stock relates to future economy and also is affected by discount rates (interest rate). The rising energy price affects the Chinese macroeconomy and pushes up the inflation rate. The cointegration obtained in this paper demonstrates that energy price has a significant effect on the stock market, which is an extension of existing literature. In equation (11), we find that in the long-term, energy price has a negative relationship with industrial value adding and the stock market. The rise in energy price affects the macroeconomy, on the one hand, and depresses the stock market on the other hand.

### 4.2. The Positive Shock of Energy Price Can Push up the Real Interest Rate, Affect Industry Productions, and Depress the Stock Market Price

 On the basis of realizing the long-term relationship among them, we want to know the response of stock prices and the macroeconomy to the short-term shock of the energy price. Based on impulse response function, we have seen that the positive shock of energy price can push-up the real interest rate, affect industry, and depress the stock market.

 The shock of energy price can affect real interest rates to some extent. In China, since 2002, the international crude oil price has risen from $20 per barrel to nearly $100 per barrel. In turn, this drives up oil related energy products and total prices. Therefore, China has the risk of cost-push inflation. The rise of international crude oil price will increase the domestic price of the refined oil and related chemical engineering products. The rise of the refined oil price can further increase the price of transportation, industrial production, and residential gas. Moreover, the rise in price of related chemical engineering products will increase the cost of plastic, rubber, chemical fiber, and other inputs. Finally, the total social price level rises. Inflation makes the supply of currency relatively insufficient. The controllable real currency on the whole will decrease. Consequently, the rise of energy price will drive up the total interest rate level.

 As a major input to industry, the rise in energy price can affect economic growth. In the Chinese industry, chemical engineering, metallurgy, and some other energy-intensive industries are seriously affected by the rise in oil price. However, following advances in technology and the upgrading of industrial infrastructure, the effect becomes less and less in the long term.

 The stock market is not completely efficient with regard to recognizing energy price. There is a phenomenon of underreaction. As a collection of energy-input companies, the rise of energy price will increase their cost and decrease their profit. If the stock market is efficient, the rise in energy price will depress the stock market immediately. But in this paper, we find that the rise in energy price will depress the stock market during a five-month lag. As the energy price in the public domain, it means there is conservatism in most investors' decisions, as acknowledged by Barberis et al. [34]. In other words, investors find it difficult to renew their views and have an underreaction to the importance of the price of energy. As a limitation in cognitive abilities, they find it hard to evaluate the importance of energy price to stock value. This conforms to the hypothesis that “information diffuses gradually” suggested by Hong and Stein [35]. 

### 4.3. The Effect on Energy Price of the Industrial Demand: Increasing Interest Rates and the Stock Market

 We have discussed earlier that the energy price will have a significant effect on industrial production, real interest rates, and the stock market. But we still want to know the effect of three shocks on energy price.

 To control the inflation caused by the rise of energy price increasing the real interest rate is very efficient. It is because, on the one hand, high interest rates change people's controllable income by increasing savings and decreasing consumption on energy relating products, and on the other hand, high interest rates will cause the capital to depreciate and reduce people's wealth, which reduces their consumption. As to industries, high interest rates increase their capital cost and depresse their investment, which reduces their energy use for relating products. Since 2007, the People's Bank of China has increased the interest rates five times and decreased the interest rate tax. Currently, a sixth increase in interest rates is being considered. It can be forecasted that the People's Bank of China will still control inflation by increasing interest rates as price increase further.

 The shock of industrial growth has a small, but long effect on energy price. So the demand from industry on energy is one of the reasons that cause a rise in the energy price. But the effect is slight.

 The bull stock market can attract a good deal of money, which reduces the currency. The Chinese stock market has risen from 1000 points to 6000 points since 2005. It has attracted a large amount of money. So after the 2008 Olympics, or when the appreciated RMB has been realized, a lot of money will be taken back from stock market. Additionally, there is risk of inflation in the near future. Consequently, the rise in energy price will be severe.

### 4.4. Energy Price, over the Longer Term, Has Better Ability to Explain the Stock Market than Interest Rates

 From the results of forecasting decomposition, we find that over the longer term, the greatest effect of three factors on stock price is energy price, interest rates, and industrial value adding. In the short term, the increase in interest rates is a *bad news* and can be perceived easily by all investors. So in the short term, the interest rates are more important than the other two factors. But in the long term, the rise in energy price will become gradually apparent to people and have an effect on people's anticipation. So it will be the more important factor in the longer term. As industries are only a part of stock market, the effects from them on the total market are limited. There is separation between the real economy and the virtual economy in China. Consequently, it has a poor explanation in explaining fluctuations in the stock market.

## 5. Conclusions and Future Work

Through the analysis of results and discussion, we can draw the following conclusions.

(1) There is a stable long-term relationship among energy price, industrial value adding, real interest rates, and stock price. In the longer term, the rise in energy price will drive up the real interest rates and have an adverse effect on industrial value adding and the stock market.
(2) The shocks in energy price have a lasting effect on real interest rates, which to some extent causes long-term inflation. The shocks in energy price have a notable effect on industrial value adding. But the effect tends to be weaker in the long term. In the short time, the shocks in energy price have an effect on the stock market.
(3) To control the rise in energy price, we should consider the macroeconomy and stock market, in addition to the international and domestic energy market. The rising industrial value adding increases the energy demand, which to some extent increases the energy price. But the rise of interest rates is favorable for controlling the energy price.
(4) The effect of energy on the stock market is hard to ignore. Through forecasting the decomposition of stock prices, we can identify that the stock price is chiefly affected by itself. What is a contradiction to the general view is that energy price and interest rates have greater effect on the stock market than industrial value adding. In the long term, energy price is even more important than interest rates. It indicates to investors that they should not only consider the interest rates but also consider the energy price for their investment.

This paper discusses the relationship between energy price and stock market at the macrolevel. The relationships between the shocks of energy price and the stock prices of various industries and oil companies remain to be researched. Meanwhile, opportunities for future research include the simulation and analysis of China economic transition and sustainable development at various levels of energy price, and the optimal design of subsidy policy for rising oil price [36]. 

## Figures and Tables

**Table 1:** The test result of unit root.

Variables	ADF test	Test type (*c*, *t*, *p*)	Critical value
IP	−2.003340	(*c*, *t*, 2)	−3.160198*
*d*IP	−1.973988	(0,0, 11)	−3.165046*
*d* ^2^IP	−4.952033	(0,0, 10)	−2.598416***
OIL	−1.808898	(*c*, *t*, 1)	−3.159780*
*d*OIL	−5.270185	(0,0, 0)	−2.593824***
*R*	−1.922089	(*c*, 0,0)	−2.585861*
*dR *	−2.995834	(0,0, 11)	−2.598416***
STOCK	−1.045074	(0,0, 0)	−2.585861*
*d*STOCK	−7.767847	(0,0, 0)	−2.593824***

*Note*. ^1^The meanings of various variables in the table are as follows: IP is industry value added discounted by price index, OIL is discounted purchase price index for fuel power, *R* stands for real interest rates, and STOCK is monthly price index of Shanghai stock market.

^2^ *c*,  *t*,   and *p* in test type stand for constant, trend, and lag orders, respectively.

^3^At three remarkable levels, when ADF value is greater than critical value, corresponding series has unit root.

^4^ ***, **, and * stand for 1%, 5%, and 10% critical levels, respectively.

^5^ *d* stands for the first differential of the variables, and *d* ^2^ stands for the second differential of the variables.

**Table 2:** The choose of lag orders in the model.

Lag	AIC	SC	Log *L*
0	−11.09981	−10.9762	420.2427
1	−20.11605	−19.49806*	774.3520
2	−20.36989	−19.2575	799.8710
3	−20.37243*	−18.7656	815.9662
4	−20.16755	−18.0664	824.2831
5	−20.30166	−17.7061	845.3124

Note: * above stand for the minimum values in the columns of AIC and SC.

**Table 3:** The series cointegration test result.

The original hypothesis	Eigenvalue	Trace statistic (*P* value)	*λ*-max statistic (*P* value)
No cointegration vector	0.606545	96.20150 (0.0000)*	73.69024 (0.0000)*
More than one cointegration vector	0.154912	22.51127 (0.2709)	13.29684 (0.4253)
More than two cointegration vector	0.089195	9.214422 (0.3459)	7.380688 (0.4451)
More than three cointegration vector	0.022945	1.833735 (0.1757)	1.833735 (0.1757)

Note: * above means that the original hypothesis is significantly rejected.

**Table 4:** The model test results.

Stability test	The coefficients of all roots are less than one So the model estimated is stable
Autocorrelation LM test	LM = 18.88 (0.2749) No series autocorrelation
Heteroskedasticity test	*χ* ^2^(240) = 231.2206 (0.6462) No heteroskedasticity
Jarque-Bera normal test	JB (8) = 7.11161 (0.5246) The residual series conform to normal distribution

**Table 5:** Generalized variance decomposition of STOCK.

Periods	S.E.	*d*IP	OIL	*R*	STOCK	Periods	S.E.	*d*IP	OIL	*R*	STOCK
1	0.029264	0.395151	0.141588	5.030712	94.43255	11	0.043981	0.342164	2.011849	3.523414	94.12257
2	0.040527	0.430799	0.470383	8.225033	90.87379	12	0.044001	0.318452	2.603733	3.671406	93.40641
3	0.041955	0.289069	1.067355	8.672081	89.97149	13	0.044018	0.29907	3.283352	3.936467	92.48111
4	0.043575	0.848855	1.11992	7.35657	90.67465	14	0.044035	0.283251	4.046391	4.29525	91.37511
5	0.043702	0.688591	0.943073	6.128618	92.23972	15	0.044049	0.270742	4.875873	4.736972	90.11641
6	0.043732	0.573093	0.803602	5.102978	93.52033	16	0.044062	0.26073	5.760365	5.249124	88.72978
7	0.043834	0.502843	0.839256	4.414149	94.24375	17	0.044074	0.253142	6.685862	5.814719	87.24628
8	0.043892	0.446533	0.952725	3.926427	94.67431	18	0.044085	0.247541	7.636951	6.422031	85.69348
9	0.043942	0.407653	1.182682	3.620365	94.7893	19	0.044096	0.243609	8.599254	7.054811	84.10233
10	0.043956	0.371269	1.533481	3.489268	94.60598	20	0.044105	0.241156	9.556565	7.699988	82.50229

Cholesky ordering: *d*IP, OIL, *R*, and STOCK.
